# Plant virus seed transmission: AGO5-mediated defense and viral counter-defense

**DOI:** 10.3389/fpls.2026.1766953

**Published:** 2026-01-21

**Authors:** Alexander A. Lezzhov, Anastasia K. Atabekova, Denis A. Chergintsev, Ekaterina A. Lazareva, Andrey G. Solovyev

**Affiliations:** A. N. Belozersky Institute of Physico-Chemical Biology, Moscow State University, Moscow, Russia

**Keywords:** AGO (Argonaute), AGO5, plant antiviral defense, plant virus, siRNA, virus meristem invasion, virus seed transmission, virus-host interactions

## Introduction

Small non-coding RNAs, such as microRNA and small interfering RNA (siRNAs), are involved in RNA silencing and play an important regulatory role in plant development, as well as in responses to different stresses and attacks by pathogens, including viruses (Lopez-Gomollon and Baulcombe, 2022). 21–24 nucleotide (nt) siRNAs originate from long double-stranded RNA (dsRNA) that is cleaved by Dicer-like (DCL) proteins; the Argonaute (AGO) family proteins bind the siRNAs and form silencing effector complexes that direct post-transcriptional gene silencing (PTGS) or transcriptional gene silencing (TGS) through complementarity with corresponding RNA targets (Vaucheret and Voinnet, 2024).

The replication of viral RNA genomes involves the formation of dsRNA replication intermediates. In virus-infected cells, DCL proteins, usually DCL4 and DCL2, cleave the viral dsRNAs into 21–22 nt siRNAs, which form complexes predominantly with AGO1 and AGO2, whose expression increases significantly in virus-infected tissues. These complexes mediate the cleavage of viral RNA as well as the suppression of translation and induction of silencing signal amplification, ultimately leading to considerable inhibition of viral infection (Lopez-Gomollon and Baulcombe, 2022). It is important to note that other AGO proteins, such as AGO5, AGO7, and AGO10, are also capable of inhibiting viral infections in plants, although to a lesser extent, and can be considered as supplementary factors in the silencing-based antiviral defense (Wang et al., 2011; Brosseau and Moffett, 2015; Brosseau et al., 2020). However, the antiviral role of these AGO proteins has not yet been clearly defined.

## Known functions of AGO5

Overall, the functions of AGO5 remain poorly understood. AGO5 has been shown to bind siRNAs of various lengths (Marchais et al., 2019; Bradamante et al., 2024), exhibiting a preference for those with a C residue at the 5′ end (5’-C-siRNAs) (Mi et al., 2008), and possess endonuclease activity (Brosseau and Moffett, 2015). Unlike AGO1 and AGO4, which are relatively uniformly expressed throughout plants, the expression of other AGO proteins is restricted to certain tissues, specific developmental stages, or stress response conditions (Martín-Merchán et al., 2023; Zaheer et al., 2024). In particular, AGO5 is specifically expressed in the cell layers L1 and L2 of the *Arabidopsis thaliana* shoot apical meristem (Bradamante et al., 2024). Furthermore, AGO5 has been detected in egg and central cells, the inner integument and the nucellus, at the chalazal pole of the ovule and in sperm cells (Borges et al., 2011; Jullien et al., 2022). After pollination, the AGO5 protein can still be detected in the embryo (Bradamante et al., 2024). It is important to note that male and female gametophytes develop from cellular descendants of the L2 layer of meristematic cells, in which AGO5 and AGO9 are expressed (Jenik and Irish, 2000). Dysfunction of AGO5 and AGO9 has been shown to lead to the malformation of female gametes, suggesting that these two AGOs have specific functions associated with the specification of gametophyte precursors (Olmedo-Monfil et al., 2010; Tucker et al., 2012), but does not affect the overall morphology of the plant (Katiyar-Agarwal et al., 2007; Roussin-Leveillee et al., 2020). The expression of AGO5 and AGO9 is assumed to be necessary to protect developing gametophytes from activated transposable elements (TEs). Both AGO5 and AGO9 effectively bind TE-derived siRNAs, then AGO9 provides TGS of TE genomic loci in the nucleus, while AGO5 provides PTGS of TE transcripts in the cytoplasm (Bradamante et al., 2024). Therefore, in non-infected plants, AGO5 is expressed and functions in meristem cells and gametophytes.

The expression level of AGO5 is undetectable or relatively low in leaves of uninfected plants; however, the AGO5 expression increases dramatically upon viral infection. Such increase has been reported for infections of potato virus X (PVX), bamboo mosaic virus (BaMV), foxtail mosaic virus, tobacco mosaic virus, cucumber mosaic virus (CMV), and grapevine pinot gris virus in *Nicotiana benthamiana*; PVX and Plantago asiatica mosaic virus in *A. thaliana*; Cymbidium mosaic virus (CymMV) in *Phalaenopsis aphrodite*; and cucumber green mottle mosaic virus in *Citrullus lanatus* (Brosseau and Moffett, 2015; Kuo et al., 2021; Tarquini et al., 2021; Ke et al., 2022; Liu et al., 2023; Tu et al., 2023). Several cell factors that bind to and positively regulate the AGO5 promoter have been identified as being upregulated during viral infections in different plant-virus systems (Kasi Viswanath et al., 2022; Ke et al., 2022; Liu et al., 2023). Notably, several specific viral factors that induce the AGO5 promoter activity have also been identified, including the TGB1 proteins of CymMV and BaMV, as well as the Odontoglossum ringspot virus movement protein (Kasi Viswanath et al., 2022; Ke et al., 2022). As demonstrated in transgenic *N. benthamiana* lines that overexpress AGO5, high levels of AGO5 accumulation are correlated with significantly reduced amounts of several analyzed viruses (Tu et al., 2023). A straightforward interpretation of these observations is that the plant defense response to viral infection involves the upregulation of AGO5 expression in infected tissue, leading to a direct antiviral effect.

This view is consistent with the data showing that some viruses can induce AGO5 degradation to promote infection. As demonstrated for CMV, the AGO5 expression is upregulated at early infection stages. Then, once a sufficient amount of the silencing suppressor 2b protein has accumulated in infected tissues, this protein binds siRNAs, thereby inhibiting their activity as well as intercellular and long-distance transport (Lopez-Gomollon and Baulcombe, 2022). Concomitantly, the 2b protein triggers the proteasomal degradation of AGO5, therefore neutralizing the AGO5 suppressive effect on virus accumulation (Takahashi et al., 2022; Tu et al., 2023). In the case of turnip mosaic virus (TuMV), the viral multifunctional silencing suppressor HC-Pro (Lopez-Gomollon and Baulcombe, 2022) is translated directly from the genomic RNA template immediately after the virus enters the cell, without the need for prior replication and transcription; on the other hand, TuMV HC-Pro directs proteasomal degradation of AGO5, similarly to CMV 2b. As a result, the AGO5 degradation is triggered at very early infection stages, and TuMV can efficiently overcome the AGO5-mediated resistance even in AGO5-overexpressing plants (Tu et al., 2023). The importance of the AGO5 degradation for viral multiplication is reinforced by the observation that *N. benthamiana* infections with CMV lacking 2b or TuMV lacking HC-Pro result in the robust AGO5 accumulation and viral infection inhibition (Tu et al., 2023).

There is no doubt that high levels of AGO5 expression have an inhibitory effect on virus accumulation in infected tissues. However, direct up-regulation of AGO5 expression by virus-encoded proteins (see above) may suggest a pro-viral function of AGO5, as discussed below.

## The role of silencing in limiting systemic infection and virus seed transmission

Using CMV as a model, it has been demonstrated that the functioning of the silencing machinery limits the transmission of viruses through seeds in *A. thaliana*. Using a CMV variant without the silencing suppressor 2b, it has been shown that the proteins DCL2, DCL4, AGO1, AGO2, RNA-dependent RNA polymerase 1 (RDR1), and RDR6 play key roles in the effective suppression of systemic viral infection. Importantly, analysis of *A. thaliana* mutant lines revealed that no effect on systemic viral transport was observed in an *ago5* line. Furthermore, no additional contribution of *ago5* to the effects of *ago1* and *ago2* was detected when studying the viral systemic infection in lines with combinations of mutations. However, when the efficiency of CMV seed transmission was studied using the same mutant lines, the virus was found to be transmitted with significantly greater efficiency in the *ago1*/*ago2*/*ago5* genotype only, and not in the single *ago1*, *ago2* and *ago5* or double *ago1*/ago2 mutants. Furthermore, it has been demonstrated that amplification of the silencing signal in plants can reduce virus transmission efficiency through seeds, as evidenced by a substantial increase in CMV seed transmission observed in the *rdr1/rdr6* genotype. This suggests that both functional AGO machinery including AGO5 and effective accumulation of secondary siRNAs through RDR activity are required for effective suppression of virus seed transmission (Liu and Ding, 2024). Data linking the production of virus-specific siRNAs to virus gametophyte entry and seed transmission are not surprising, as the defense mechanism that limits the systemic spread of viruses is based on the long-distance transport of virus-specific siRNAs, which are produced at sites of virus infection, to the upper, non-infected plant parts via the phloem. Importantly, the systemic transport of these siRNAs is assumed to be faster than that of viral genomes. As a result, an antiviral state is established in cells of systemic tissues before viruses reach them, providing virus resistance to these tissues and suppressing virus systemic spread (Malavika et al., 2023; Elvira-González et al., 2025). This mechanism likely plays a central role in establishing an antiviral state in gametophytes, and we hypothesize that AGO5 is a key element involved in modulation of the efficiency of virus seed transmission.

## Hypotheses

Based on the available data, we hypothesize that AGO5 plays a central role in limiting virus seed transmission and can be manipulated by plant-infecting viruses to inhibit the AGO5-dependent defense mechanism.

**Figure 1:**
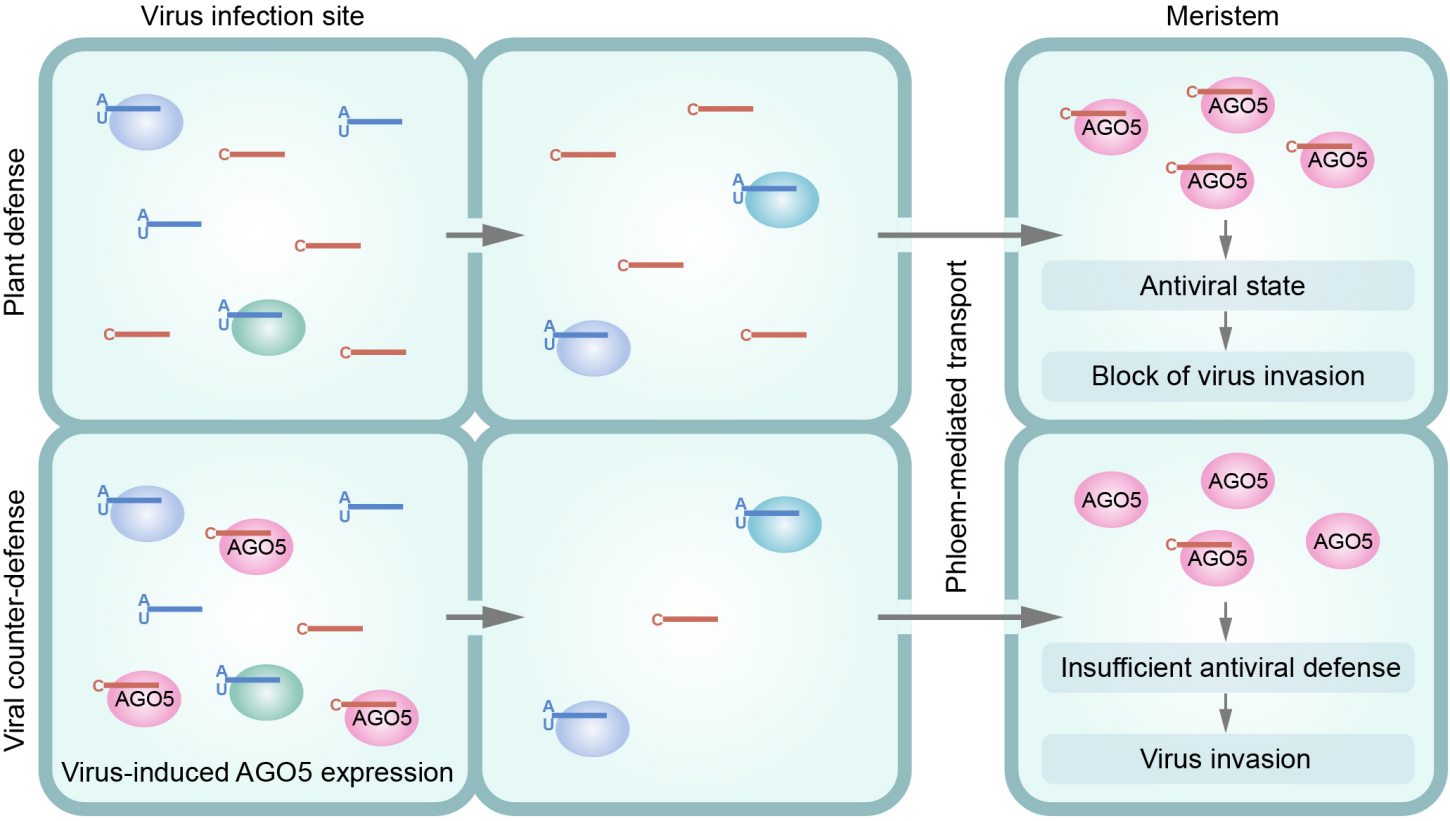
Proposed model of AGO5-mediated control of virus entry into meristem tissues (top) and viral counter-defence to AGO5-mediated block of virus meristem invasion (bottom). The meristem-specific AGO5 is depicted as a red oval; AGO proteins expressed in somatic tissues are shown as ovals of other colors. Virus-specific siRNAs produced at infection sites are shown with an indication of their 5’-nucleotide residues. 5’-G-siRNAs are not considered, as AGO protein(s) that bind such siRNAs are not yet identified. The model is based on two premises: (1) the currently accepted view that siRNAs are capable of cell-to-cell and systemic transport in plants, but lose or strongly reduce their mobility when bound by AGO proteins, and (2) data showing that AGO proteins expressed in somatic cells predominantly bind 5’-A/U-siRNAs, while AGO5 expressed in the meristem and gametophytes preferentially binds 5’-C-siRNAs. According to the model, somatic cells-specific AGO proteins bind viral 5’-A/U-siRNAs at infection sites or in cells that siRNAs pass through during transport. However, binding of 5’-C-siRNAs on the way to the meristem is minimal and, consequently, 5’-C-siRNAs are efficiently delivered to the meristem. AGO5 binds 5’-C-siRNAs in the meristem, forming effector complexes that establish an antiviral state that blocks virus invasion into the meristem and precludes further virus seed transmission. As a viral counter-defense against the AGO5-mediated block of meristem invasion, viruses induce upregulation of AGO5 expression at infection sites. This leads to the binding of 5’-C-siRNAs by AGO5 in infected cells and the inhibition of 5’-C-siRNAs cell-to-cell and systemic transport. As a result, a minimal amount of 5’-C-siRNAs, if any, are delivered to the meristem. There, the antiviral state cannot be established, thereby allowing virus invasion into the meristem and further seed transmission.

In plants, systemic transport of siRNAs occurs simplistically. This means that the virus-specific siRNAs pass through different types of cells on its way from virus-infected tissues to the apical meristems. AGO proteins, which preferentially bind siRNAs with A or U residues at their 5’-ends (5’-A/U-siRNA), are expressed in somatic cells. Based on several indirect lines of evidence, it is currently assumed that siRNAs lose, or at least strongly reduce, their ability to move cell to cell once they are bound by AGO proteins (Voinnet, 2022). Consequently, the population of siRNAs moving through the plant becomes depleted of the 5’-A/U-siRNAs due to their binding by AGO proteins in the cells they pass through. As a result, 5’-G/C-siRNAs are the class of virus-specific siRNAs that are preferentially delivered systemically to the meristem tissues (Devers et al., 2020; Voinnet, 2022). To date, a plant AGO that binds 5’-G-siRNAs has not been identified (Voinnet, 2025). On the other hand, AGO5 is predominantly expressed in meristems and preferentially binds 5’-C-siRNAs (Jenik and Irish, 2000; Mi et al., 2008). Therefore, we hypothesize that AGO5 expressed in the meristem perceives the systemically transported virus-specific 5’-C-siRNAs (**Figure 1**) and suggest that AGO5 is necessary for establishing an antiviral state in the gametophytes and inhibiting the virus invasion into the generative tissues.

To counteract this defense mechanism that prevents virus invasion of the gametophyte, viruses can specifically reduce the amount of 5’-C-siRNAs that reaches meristems by increasing AGO5 expression in virus-infected tissues. As a result, the virus-specific 5’-C-siRNAs are bound by AGO5 at the sites of their biogenesis, preventing or considerably inhibiting their systemic transport. Consequently, only small amounts of 5’-C-siRNAs, if any, reach the meristem. This precludes the establishment of a full antiviral state, allowing the virus to invade the gametophytes (**Figure 1**).

The hypothesized AGO5-specific antiviral defense mechanism that limits virus seed transmission and its counteraction by viruses are consistent with the majority of available experimental data, remaining however plausible but untested interpretations. It should be also noted that the proposed model may not apply to all plant-virus interactions. Therefore, further research is needed to provide more direct evidence required to fully elucidate the role of AGO5 in the plant antiviral defense.
